# Targeting Mitochondrial Quality Control for the Treatment of Triple-Negative Breast Cancer: From Molecular Mechanisms to Precision Therapy

**DOI:** 10.3390/biom15070970

**Published:** 2025-07-05

**Authors:** Wanjuan Pei, Ling Dai, Mingxiao Li, Sihui Cao, Yili Xiao, Yan Yang, Minghao Ma, Minjie Deng, Yang Mo, Mi Liu

**Affiliations:** 1 Medical College, Hunan University of Chinese Medicine, Changsha 410208, China; wanjuanpei@stu.hnucm.edu.cn (W.P.); lingdai@stu.hnucm.edu.cn (L.D.); ellie@stu.hnucm.edu.cn (M.L.); yili_xiao@stu.hnucm.edu.cn (Y.X.); yangyan1@hnzyydxxyh.wecom.work (Y.Y.); minghaoma@stu.hnucm.edu.cn (M.M.); 202308010404@stu.hnucm.edu.cn (M.D.); 2 College of Acupuncture and Moxibustion, Hunan University of Chinese Medicine, Changsha 410208, China; 20242115@stu.hnucm.edu.cn

**Keywords:** TNBC, mitochondrial quality control, immune evasion, metabolic reprogramming, maintenance of stemness

## Abstract

Breast cancer is the leading threat to the health of women, with a rising global incidence linked to social and psychological factors. Among its subtypes, triple-negative breast cancer (TNBC), which lacks *estrogen receptor (ER)*, *progesterone receptor (PR)*, and *human epidermal growth factor receptor 2 (HER2)* expression, is highly heterogeneous with early metastasis and a poor prognosis, making it the most challenging subtype. Mounting evidence shows that the mitochondrial quality control (MQC) system is vital for maintaining cellular homeostasis. Dysfunction of the MQC is tied to tumor cell invasiveness, metastasis, and chemoresistance. This paper comprehensively reviews the molecular link between MQC and TNBC development. We focused on how abnormal MQC affects TNBC progression by influencing chemoresistance, immune evasion, metastasis, and cancer stemness. On the basis of current studies, new TNBC treatment strategies targeting key MQC nodes have been proposed. These findings increase the understanding of TNBC pathogenesis and offer a theoretical basis for overcoming treatment challenges, providing new research angles and intervention targets for effective precision therapy for TNBC.

## 1. Introduction

According to the global cancer statistics released by the Global Cancer Observatory (GCO) in 2022, the incidence of breast cancer ranks second globally, only after lung cancer, with a mortality rate as high as 6.9% [1]. This poses a serious threat to the health of women worldwide. TNBC, as the most aggressive subtype of breast cancer, has higher incidence and mortality rates than other subtypes. It lacks the three targets of *ER*, *PR*, and *HER2*, and accounts for approximately 15% of all breast cancer cases [2]. It is worth noting that TNBC has particularly prominent aggressive biological behavior. Epidemiological data show that about 46% of TNBC patients will develop distant metastasis, with a median survival of only 13.3 months after metastasis. The metastasis often involves organs such as the brain and viscera, and the recurrence rate within 3 years after surgery is as high as 25% [3]. This high risk of metastasis and recurrence makes TNBC one of the most challenging types of cancer to treat clinically.

The molecular heterogeneity of TNBC is the core of its therapeutic challenge. The classification methods vary depending on the population studied. This heterogeneity results in traditional treatment strategies being unable to cover all patients, and most patients respond poorly to existing therapies. The current treatment of TNBC mainly relies on surgery, chemotherapy, and radiotherapy [4]. However, it still faces multiple challenges. For example, TNBC has a high resistance to anthracyclines, taxanes, and other chemotherapeutic drugs. The mechanisms involve the coordinated action of multiple factors, such as drug efflux mediated by ABC transporters, dormancy of CSCs, hypoxic microenvironment, and evasion of apoptosis [5]. Moreover, although TNBC has a relatively high immunogenicity, the objective response rate of monotherapy with *programmed death-1 (PD-1)/programmed death-ligand 1 (PD-L1)* inhibitors is less than 25% [6]. Additionally, the survival benefit from the combination of immune checkpoint inhibitors (ICIs) is limited [6]. Due to the lack of expression of *ER*, *PR*, and *HER2*, TNBC is unresponsive to endocrine therapy and *HER2*-targeted drugs [7]. Although Poly (ADP-ribose) polymerase (PARP) inhibitors have shown some efficacy in patients with *Breast cancer susceptibility gene (BRCA)* mutations, they are only applicable to a minority of subtypes [8].

In recent years, it has been confirmed that the abnormality of MQC is an important driving factor for the malignant phenotype of TNBC. Disruption of MQC can mediate chemoresistance and distant metastasis through pathways such as enhancing OXPHOS metabolism, inhibiting mitophagy, and promoting epithelial–mesenchymal transition (EMT) [9,10]. Moreover, the imbalance of MQC can lead to upregulation of *PD-L1* expression and mitochondrial translocation, thereby inducing T-cell exhaustion and immune evasion [11]. Based on the above mechanisms, targeting key nodes of MQC has become a new paradigm for reversing the therapeutic challenges of TNBC. At the same time, combination therapies and novel targeted drugs have also shown significant potential in preclinical studies. In the future, with the advancement of molecular classification techniques and in-depth multiomics research, precision treatment strategies are gradually becoming mainstream. Exploring the interactions between MQC and other signaling pathways, as well as the clinical translation of novel targeted drugs, may become potential directions for breaking through the treatment of TNBC.

## 2. Triple-Negative Breast Cancer

TNBC is a subtype of breast cancer characterized by high malignancy, propensity for recurrence, and poor prognosis [4]. Currently, the diagnosis of TNBC mainly relies on immunohistochemical (IHC) testing, which confirms the absence of expression of *ER*, *PR*, and *HER2* to make the diagnosis [8]. However, this diagnostic method is limited in that it fails to provide detailed information on tumor heterogeneity, which is crucial for precision treatment. Recent studies have shown that radiomics has potential application value in the diagnosis and heterogeneity analysis of TNBC. Lin Jiang et al., [10], using the Fudan classification (FUSCC classification) as the gold standard, analyzed the data of 860 breast cancer patients (including 246 TNBC patients) and found that the combined model of radiomics and IHC staining data had a higher area under the receiver operating characteristic curve (AUC) in the basal-like immune-suppressed (BLIS) subtype than the individual radiomics and IHC models . This finding provides a new idea for non-invasive precise subtyping.

The “triple-negative” feature of TNBC differentiates it from luminal and *HER2*-positive breast cancers, rendering endocrine therapy and anti-*HER2* treatment ineffective for TNBC [4]. This significantly limits the treatment options for TNBC. Therefore, research on TNBC subtype classification based on molecular characteristics and differences in signaling pathways is of great significance. At present, there are three internationally influential TNBC classification methods: (1) FUSCC classification [12,13], (2) Lehmann classification system [14,15,16], and (3) Burstein classification [17,18,19]. These classification methods, by more finely dividing TNBC subtypes, lay the foundation for providing more precise treatment plans for patients. Given the particularity and therapeutic challenges of TNBC, researchers are committed to identifying new therapeutic targets, such as Mucin 1 (MUC1) [20] and Nudix-type motif 5 (NUDT5) [21]. This article will systematically summarize the relevant targets of MQC-targeted therapy for TNBC, providing a reference for the therapeutic prospects of TNBC.

## 3. Mitochondrial Quality Control

**Figure 1:**
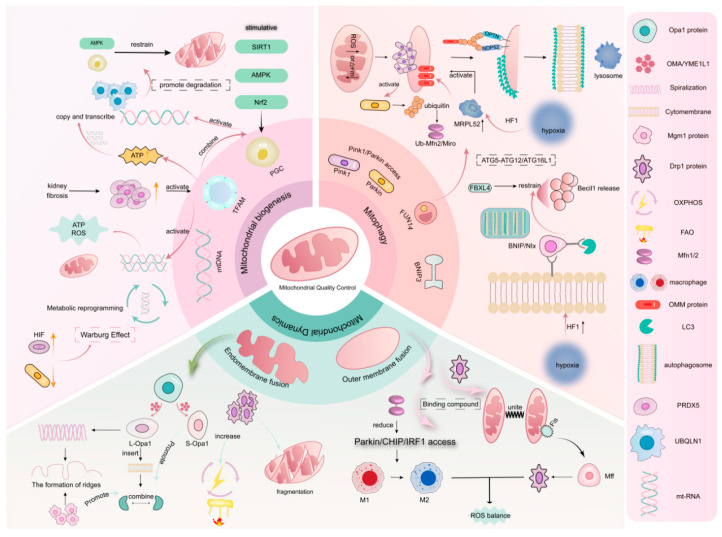
This schematic diagram comprehensively illustrates the regulatory network of mitochondrial quality control, including the regulation of *mtDNA* during mitochondrial biogenesis by the *SIRT1–AMPK–PGC1α–TFAM* signaling axis; the activation mechanisms of two key autophagy pathways, PINK1/Parkin and BNIP3/NIX; and the dynamic balance between mitochondrial fusion mediated by MFN1/2-OPA1 and Drp1-Mff/Fis1-regulated fission. The diagram details important molecular events, such as UBQLN1-mediated degradation of *PGC-1α* and Parkin-mediated ubiquitination of MFN2, highlighting the pathophysiological importance of these pathways in tumor metabolic reprogramming and neurodegenerative diseases. The image is based on the Adobe Illustrator (2020) platform.

Mitochondria, as the energy factories and metabolic regulation centers of the cell, often participate in the occurrence and progression of various diseases when their functions are disrupted. In recent years, an increasing number of studies have shown that MQC is not only involved in energy metabolism and the production of important substances, but also plays a significant role in processes such as apoptosis, oxidative stress, immune regulation, and metabolic reprogramming. It can also shape the tumor microenvironment. Currently, it has become a major research hotspot in the treatment of TNBC [4,22]. The MQC system, through processes such as mitochondrial biogenesis, autophagy, and dynamics, forms a dynamic and sophisticated regulatory network to maintain the stability of mitochondrial structure and function. A detailed exploration of the occurrence and specific mechanisms of MQC (Figure 1) helps to understand the onset of diseases and provides a new perspective for the intervention and treatment of TNBC.

### 3.1. Mitochondrial Biogenesis

Mitochondrial biogenesis, as the final step in MQC, mainly regulates cellular homeostasis by increasing the number of mitochondria in the cytoplasm [23]. Mitochondria have their own set of DNA, namely *mitochondria DNA (mtDNA)*, where *mitochondrial transcription factor A (TFAM)* plays a key regulatory role in the replication and transcription of *mtDNA*. When cells are under stress, *TFAM* can clear *mtDNA* through the autophagy pathway, thereby avoiding its induction of inflammatory responses [24]. Studies have found that peroxiredoxin 5 (PRDX5) can enhance the function of *TFAM*, thereby promoting the production of ATP in mitochondria and the recovery of *mtDNA* copy number, ultimately alleviating respiratory suppression in diseases [25]. Secondly, *peroxisome proliferator-activated receptor gamma coactivator 1α (PGC-1α)*, as a transcriptional coactivator, can regulate the expression of mitochondrial proteins. For example, *PGC-1α* controls the transcription of *mtDNA* by binding to the *TFAM* promoter and promotes mitochondrial biogenesis. It also coactivates nuclear respiratory factor 1 (Nrf1) and Nrf2 to promote the expression of genes related to the antioxidant system [26]. Therefore, studying the biogenesis mediated by the *PGC-1α/TFAM* pathway may be of great significance for the occurrence and development of diseases. Fan et al. [27,28] found through experiments that *PGC-1α* can serve as a target for inhibiting mitochondrial gene expression, thereby intervening in the further metastasis of TNBC to improve prognosis. AMP-activated protein kinase (AMPK) can act as an activator to enhance the expression of *PGC-1α*, promote biogenesis, and reduce mitochondrial damage, thereby inhibiting cell death and exerting a certain protective effect on organ systems to improve patient survival.

It is worth noting that mitochondria, as the main regulators of pyroptosis, release large amounts of reactive oxygen species (ROS) when damaged, thereby triggering a large number of inflammatory responses. The damage to mitochondria is mainly due to the disruption of processes such as mitochondrial biogenesis that provide energy demands. Therefore, mitochondrial biogenesis is closely related to ROS. Studies have shown that [29] the levels of certain protein structures, such as ubiquilin 1 (UBQLN1), can regulate the production and degradation of *PGC-1α*, leading to a reduction in mitochondrial biogenesis. In this process, the decrease in ROS levels also plays an important role. It can be seen that *PGC-1α* plays an important, pivotal role in regulating mitochondrial function. Similarly, to adapt to environmental changes, tumor cells generally adopt the process of metabolic reprogramming to meet the growth needs of cells, that is, the abnormal initiation of glycolysis known as the “Warburg effect” [30]. This change often optimizes the biosynthetic process and improves energy utilization efficiency by inducing the expression of related factors or proteins [31]. This suggests that by acting on related genes and metabolic pathways to regulate mitochondrial biogenesis and antioxidant defense processes, it may be possible to effectively intervene in the tumor’s survival environment.

### 3.2. Mitophagy

Mitochondrial autophagy, an adaptive metabolic response that clears damaged or excess mitochondria, prevents ROS accumulation and cell death [32]. This type of autophagy also holds an important position in the proliferation of cancer cells. For example, when FUN14 domain-containing protein 1 (FUNDC1) mediates Parkin-independent mitophagy, it restores autophagosome biogenesis by promoting the formation of the ATG5-ATG12/ATG16L1 complex. However, it also acts as a tethering protein of the mitochondrial–*ER* contact component, altering its structure to meet the energy demands of rapid tumor cell proliferation [33], and targeting this pathway can have tumor-suppressive effects by inducing ROS-related mitochondrial changes. Conversely, high FUNDC1 expression is linked to poor breast cancer prognosis [34].

In recent breast cancer studies, Ursolic acid (UA) was found to enable *transcription factor EB (TFEB)* nuclear entry in a partial mammalian target of rapamycin (mTOR)-dependent manner and prevent its ubiquitination. Once in the nucleus, *TFEB* activates the mitochondrial autophagy–lysosome pathway to remove damaged mitochondria, reducing inflammatory factors and showing therapeutic potential [35].

#### 3.2.1. PINK1/Parkin Signaling Pathway

The PTEN induced kinase 1 (PINK1)/Parkin pathway, a key mechanism for mitochondrial autophagy, is activated when the mitochondrial membrane potential (Δψm) is dissipated or under oxidative stress, leading to PINK1 accumulation. However, mild oxidative stress may not trigger this pathway [36]. Once activated, PINK1 phosphorylates and ubiquitinates mitochondrial surfaces, recruiting Parkin to initiate autophagy. Parkin ubiquitinates outer mitochondrial membrane proteins, marking mitochondria for degradation via autophagosome–lysosome fusion. Additionally, PINK1/Parkin ubiquitinates the mitochondrial transport protein Miro, halting mitochondrial movement. Under oxidative stress, this pathway also promotes the formation of mitochondrial-derived vesicles (MDVs) for mitochondrial repair [37].

The ubiquitin–proteasome system (UPS), particularly the E3 ubiquitin ligase Parkin, plays a significant role in mitochondrial autophagy. Parkin mediates the ubiquitination of mitofusin 2 (MFN2) on the outer mitochondrial membrane (OMM), especially after PINK1 phosphorylates MFN2. This process reduces mitochondrial fusion, increases fission, and promotes autophagosome formation [38]. The downregulation of another ubiquitin ligase CRL4CUL4A/DDB1 can also significantly enhance this process. Since it can maintain cellular energy balance, this process can also inhibit the proliferation of cancer cells [39].

In cancer cells, hypoxia-induced HIF-1 upregulates the mitochondrial ribosomal protein (MRPL52), promoting PINK1/Parkin-dependent mitochondrial autophagy to clear ROS and protect cancer cells [40]. Similarly, Fanconi anemia complementation group C (FANCC) overexpression enhances this pathway, reducing necroptosis. FANCC interacts with TANK-binding kinase 1(TBK1), which stabilizes PINK1 and facilitates Parkin recruitment [41].

#### 3.2.2. BNIP Signaling Pathway

BCL2-interacting protein 3 (BNIP3) and its homolog NIX/BNIP3L are BH3-only domain-containing proteins critical for receptor-mediated mitophagy, a process that is independent of mitochondrial ubiquitination. These proteins function as tumor suppressors and are tightly linked to mitochondrial homeostasis. Upon binding to BCL2, BNIP3/NIX liberates Beclin1, increasing its availability for autophagosome formation [42]. Notably, BNIP3 stabilization is regulated by calnexin (CANX) overexpression, which activates mitogen-activated protein kinase (MAPK) signaling to amplify protective mitophagy. MAPK, as a direct effector of oncogenic signaling pathways, plays a key role in the growth, metastasis, drug resistance, and stemness of tumor cells. Studies have found that ROS-mediated mitophagy is detrimental to the synthesis of hormones such as progesterone and can even affect the body’s hormone levels [43,44].

As a downstream effector of HIF-1α, BNIP3 is essential for hypoxia/reoxygenation (H/R)-induced mitophagy. HIF-1α knockout attenuates mitophagy, exacerbating ROS accumulation and apoptosis in acute kidney injury (AKI), whereas BNIP3 overexpression rescues this phenotype, highlighting its cytoprotective role [45]. This suggests that BNIP3 can provide an advantage for the survival and progression of cancer cells by promoting the autophagy process and eliminating damaged mitochondria, which is also one of the sources of intracellular ROS. This argument has been confirmed in relevant experiments [46].

NIX, a paralog of BNIP3, is indispensable for mitochondrial clearance during erythroid differentiation. Enhancing NIX-mediated mitophagy accelerates erythroid maturation [47]. Both BNIP3 and NIX are OMM proteins whose expression is induced by HIF-1α. These proteins interact with core autophagy components (e.g., WIPI2/3) through LC3-interacting regions (LIRs), facilitating autophagosome engulfment of damaged mitochondria and ROS mitigation. Furthermore, WIPI family proteins engage with the minimal essential region (MER) required for autophagosome formation. Intriguingly, F-box and leucine-rich repeat protein 4 (FBXL4) acts as a key suppressor of NIX/BNIP3-dependent mitophagy, whereas HIF-1α drives BNIP3 upregulation under hypoxia [48,49].

### 3.3. Mitochondrial Dynamics

#### 3.3.1. Mitochondrial Fusion

Mitochondrial dynamics, primarily comprising mitochondrial fusion and fission, are crucial for maintaining the structural and functional integrity of mitochondria. In TNBC, mitochondrial fusion is a highly coordinated dynamic process involving both inner and outer membrane fusion, with the participation of multiple proteins. This process plays key roles in material transport, population homogenization, and oxidative phosphorylation, significantly contributing to cellular functional regulation and adaptability [50]. Mitochondrial fusion has emerged as a novel therapeutic target for various diseases.

The optic atrophy 1 (OPA1) protein is central to inner mitochondrial membrane (IMM) fusion [51]. OPA1 has enzymatic activities, including GTPase and metalloprotease functions. Under physiological conditions, OPA1 is hydrolyzed in the mitochondrial matrix by the metalloproteases OMA1 and YME1L1 into long isoforms (L-OPA1) anchored to the IMM and soluble short isoforms (S-OPA1) localized in the intermembrane space. L-OPA1 mediates initial membrane contact through its hydrophobic domain inserted into the IMM and has recently been implicated in tumor cell metabolism and proliferation [52]. S-OPA1, which is confined to the intermembrane space, interacts with L-OPA1 to facilitate IMM fusion [53].

To increase fusion efficiency, after Opa1 is hydrolyzed, it forms a helical structure under the action of GTPase, which draws adjacent IMMs closer and generates membrane protrusions to enable docking. These junctional sites may also influence crista formation [54]. OMM fusion is driven primarily by Mfn1/Mfn2, which mediate trans-interactions between adjacent mitochondria. GTP hydrolysis facilitates the formation of “tethered complexes,” ultimately completing fusion [55]. Recent studies [56] have demonstrated that the downregulation of Mfn1 can trigger the Parkin/CHIP/IRF1 pathway to enhance the expression of IL-12, which plays a positive role in the activation of immune responses.

Additionally, the mitochondrial metalloprotease OMA1 critically regulates fusion dynamics [57]. During membrane depolarization, OMA1 collaborates with yeast mitochondrial escape 1-like protease (YME1L) to cleave OPA1, shifting the L-OPA1/S-OPA1 ratio and modulating fusion capacity. Furthermore, the OPA1 homolog Mgm1 protein also has GTPase activity. Its unique structural features enable the inner mitochondrial membranes to fuse with each other under the energy provided by GTPase hydrolysis. It also induces membrane bending through multimodal polymerization and binding to multiple membrane sites to adapt to mitochondrial membrane fusion and cristae formation [58]. It is worth mentioning that studies have already found that [59] the roles of key proteins such as Opa1 and OMA in mitochondrial fusion may provide new targets for the treatment of TNBC. By regulating the activity or expression levels of these proteins, it may be possible to affect the metabolic reprogramming and drug resistance of TNBC cells.

#### 3.3.2. Mitochondrial Division

Mitochondrial fission, a crucial aspect of mitochondrial dynamics. This process plays essential roles in maintaining mitochondrial network morphology, regulating organelle size, and participating in cellular metabolic regulation and stress responses [60]. Dynamin-related protein 1 (Drp1), encoded by the *DNM1L* gene, serves as a pivotal GTPase that drives mitochondrial fission. The phosphorylation of Drp1 at Ser616, which is catalyzed by various protein kinases, constitutes a critical regulatory mechanism for fission initiation [61]. Through GTP hydrolysis, Drp1 releases the substantial energy required for mitochondrial membrane constriction and subsequent organelle separation.

During fission initiation, cytoplasmic Drp1 undergoes activation and subsequently translocates to predetermined fission sites under the regulation of specific signaling proteins. At these sites, Drp1 oligomerizes to form annular structures encircling mitochondrial membranes, facilitating membrane constriction through mechanical force generation. Notably, this process is sensitive to changes in the mitochondrial metabolic status and membrane potential. Studies have found that [62] the GTPase activity and changes in membrane potential involved in this process may also affect the proliferation and survival of TNBC cells, thereby intervening in the progression of the disease. The mitochondrial fission machinery involves the coordinated action of mitochondrial fission factor (Mff) and mitochondrial fission 1 protein (Fis1), both of which are localized to the outer mitochondrial membrane but play distinct functional roles. Mff primarily facilitates Drp1 recruitment and oligomerization at fission sites through direct protein–protein interactions, with substantial evidence confirming its critical role in precise Drp1 localization [63]. In contrast, Fis1 functions as an essential membrane protein for mitochondrial and peroxisomal fission in both mammalian and yeast cells, serving as a molecular scaffold that assists in Drp1 recruitment and facilitates oligomeric ring assembly [64]. Experimental data suggest synergistic cooperation between Mff and Fis1 in promoting Drp1-mediated fission, with functional complementarity observed under varying physiological conditions [65]. Emerging research highlights the metabolic implications of Drp1-regulated fission, particularly its capacity to increase the oxidative phosphorylation (OXPHOS) efficiency and FAO capacity. Furthermore, Drp1 has regulatory effects on the homeostasis of cellular ROS and the functional modulation of macrophages. These mechanisms collectively influence tumor metabolic reprogramming and antitumor immunity, suggesting potential therapeutic applications in oncology [66].

## 4. Relationship Between MQC and TNBC

Mitochondria, as the “power source” of the cell, exist in the cytoplasm through the process of endosymbiosis with bacteria. Its main functions revolve around energy supply and biosynthesis [67], and it also plays an important role in the proliferation, metastasis, and invasion of TNBC cells. In mitochondria, the TCA cycle is the core hub of energy metabolism, where intermediates generated from nutrients produce reducing equivalents through oxidation and key metabolites for biosynthetic reactions [68]. Based on this, the OXPHOS metabolic pathway transports electrons through the electron transport chain (ETC) in the inner membrane to generate ATP. Given that the proteins and complexes in this pathway can serve as targets for cancer therapy, a large number of related inhibitors have been developed in recent years [69].

**Figure 2:**
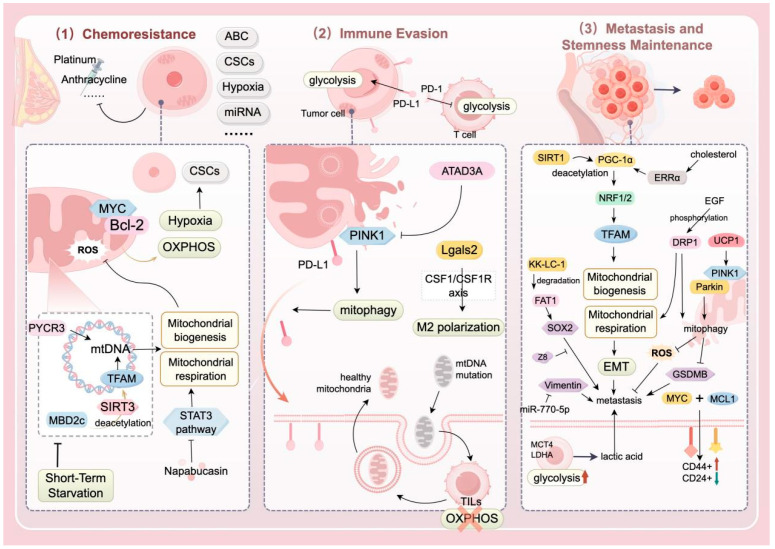
Mechanistic network of MQC abnormalities driving the malignant phenotype of TNBC. This diagram integrates the link between MQC imbalance and key TNBC pathological processes (chemoresistance, immune evasion, metastasis, and stemness maintenance). (1) Chemoresistance mechanisms such as mitochondrial metabolic remodeling (enhanced OXPHOS and abnormal *mtDNA* transcription), CSC enrichment (via *MYC/MCL1* activation), ROS accumulation, and targeted strategies (short-term starvation with chemotherapy and the STAT3 inhibitor napabucasin); (2) immune evasion processes such as upregulated *PD-L1* (from *ATAD3A*-induced mitophagy inhibition), mitochondrial transfer (EV-mediated healthy mitochondria uptake), T-cell metabolic suppression (impaired glycolysis), and interventions (metformin with 2–DG and *Lgals2/CSF1R* axis inhibition); (3) metastasis and stemness maintenance involving enhanced EMT (*SIRT1–PGC–1α*/OXPHOS axis-driven), metabolic heterogeneity (reverse Warburg effect), stem cell traits (*ALDH+/SOX2* activation), and targeted therapies (Z8-mediated *FAT1*-Hippo pathway inhibition). ROS and hypoxia, depicted throughout, synergistically promote malignant phenotypes. The key molecules and pathways are described as follows: *mtDNA*; ABC transporters; CSCs; TILs; *PD-L1*; *ATAD3A*; *Lgals2*; KK-LC-1; DRP1; UCP1; napabucasin (a STAT3 pathway inhibitor); Z8 (a KK-LC-1 pathway blocker); MBD2c; *PYCR3*; *TFAM*; *SIRT3*; MCL1; *LDHA*; and *MCT4*. The figure was drawn on the FigDraw (https://www.figdraw.com/static/index.html#/paint_user_home, accessed date: 5 May 2025) platform.

As our understanding of cell death modes continues to evolve, mitochondria have once again been revealed to be closely related to biological processes such as ROS generation, ferroptosis, apoptosis, and calcium ion homeostasis [70]. This means that the important role of mitochondria in the progression of TNBC has once again been consolidated. Numerous studies have shown that when MQC is impaired, mitochondria are highly susceptible to oxidative stress damage, which induces ROS production and thereby promotes lipid peroxidation, mediating a form of cell death characterized by iron accumulation and lipid peroxidation—ferroptosis [71]. In addition, during apoptosis, the increase in mitochondrial outer membrane permeability (MOMP) releases cytochrome C, which promotes the formation of apoptotic bodies, the cleavage and activation of caspase-9, and the activation of caspase-3 [72]. So, how does MQC affect the survival and death of TNBC cells? In the following text, we further explore this question from three main aspects: chemoresistance, immune evasion, metastasis, and stemness maintenance (Figure 2 and Table 1).

### 4.1. Impact of MQC Abnormalities on the Progression and Prognosis of TNBC

#### 4.1.1. Chemotherapy Resistance

Triple-negative breast cancer is characterized by high recurrence rates, aggressive behavior, and poor survival outcomes [73,74]. Systemic adjuvant–neoadjuvant chemotherapy (e.g., anthracyclines, taxanes, and platinum-based agents) remains the primary treatment for newly diagnosed patients [75]. However, chemotherapy, which is currently the sole systemic option for TNBC, faces considerable limitations, including severe side effects and inevitable chemoresistance [76]. Overcoming chemoresistance is critical to improving prognosis and extending survival [73]. The proposed mechanisms of chemoresistance, as summarized by Nedeljković M et al. [5], involve multifactorial cooperation, including: (1) drug efflux via ATP-binding cassette (ABC) transporters, (2) survival of quiescent CSCs post-chemotherapy, (3) hypoxic tumor microenvironments, (4) apoptosis evasion, (5) unique *miRNA* expression profiles, and (6) intratumoral heterogeneity.

Accumulating evidence highlights mitochondria as central regulators of development, stem cell fate, and metabolic reprogramming [77]. Mitochondria-driven metabolic remodeling increases tumor cell adaptation to hypoxia and nutrient deprivation, directly or indirectly promoting survival and progression [78]. Specifically, TNBC CSCs exhibit a preference for OXPHOS, which is linked to mitochondrial biogenesis and quality control [10,79]. MYC transcription factors and antiapoptotic Bcl-2 family proteins (e.g., MCL-1) are enriched in chemoresistant TNBC cells, where they cooperatively enhance OXPHOS, activate hypoxia pathways, and sustain CSC populations to drive chemoresistance [10].

Mitochondria, as the central hub of cellular metabolism, depend on genetic components encoded by both the nuclear genome and *mtDNA*. Consequently, *mtDNA* mutations or transcriptional defects are key drivers of chemotherapy resistance in TNBC cells. Yijie Hao et al. [80] reported that methyl-CpG-binding domain protein 2c (MBD2c) is highly enriched in the *mitochondrial noncoding region (NCR)* of TNBC cells, where it activates mitochondrial *TFAM* via *SIRT3*-mediated deacetylation, increasing *mtDNA* transcription and respiration. MBD2c inhibition increases cisplatin sensitivity, implicating *mtDNA* regulation in chemoresistance [80].

Although upstream mechanisms (*mtDNA* transcription, respiration) are well characterized, downstream responses to mitochondrial dysfunction, such as ROS accumulation and damaged mitochondrial clearance, remain underexplored. The *STAT3* inhibitor napabucasin enhances paclitaxel sensitivity in TNBC by suppressing *STAT3* signaling, impairing mitochondrial complex I activity and ATP production, and inducing ROS accumulation [81]. Additionally, the natural compound Guangsangon E (GSE), which is isolated from mulberries, inhibits TNBC growth by inducing mitochondrial dysfunction and mitophagy, leading to ROS overload. These findings highlight mitochondrial-targeted strategies to combat TNBC chemoresistance.

#### 4.1.2. Immune Evasion

TNBC, characterized by high *PD-L1* expression and abundant tumor-infiltrating lymphocytes (TILs), presents immunogenic features that make it a potential candidate for immune checkpoint inhibitor (ICI) therapy [82]. However, clinical data reveal the limited efficacy of anti-*PD-L1* monotherapy, with objective response rates less than 25% [6], and combined chemotherapy strategies fail to significantly improve overall survival [83]. Recent studies have implicated dysregulated MQC in tumor metabolic reprogramming, redox homeostasis, and immune evasion, with MQC abnormalities closely linked to TNBC immunotherapy resistance. In TNBC, cancer cells upregulate *PD-L1* to suppress T-cell effector functions, facilitating immune escape [11]. Furthermore, *PD-L1/PD-1* interactions enhance tumor cell glycolytic metabolism while inhibiting T-cell glycolysis, conferring survival advantages in nutrient-deprived tumor microenvironments [84].

Jernej Repas et al. [85] demonstrated that MDA-MB-231 cells treated with metformin and 2-deoxy-D-glucose exhibit morphologically intact mitochondria with increased volume and enhanced mitochondrial quality. Notably, this combination significantly suppresses glycosylated *PD-L1*, yielding potent antitumor effects, and their research further confirms the important role of *PD-L1* in the immune evasion process of TNBC tumor tissue. However, the mechanistic link between mitochondrial quality control and glycosylated *PD-L1* remains unexplored [85]. Additionally, paclitaxel-resistant TNBC cells upregulate *ATPase family AAA domain-containing protein 3A (ATAD3A)*, which inhibits PINK1-dependent mitophagy, reduces mitochondrial *PD-L1* localization, and promotes plasma membrane accumulation of *PD-L1* to amplify immune evasion [86]. This finding underscores the interplay between chemoresistance and immune escape in TNBC, warranting further exploration of this mechanistic network.

In TNBC, MQC dysregulation also modulates *mtDNA* dynamics and intercellular mitochondrial transfer to drive immune evasion. Intriguingly, TNBC cells utilize bidirectional mitochondrial transfer, exporting *mtDNA*-mutated mitochondria to TILs via extracellular vesicles (EVs) while importing healthy mitochondria from T cells, to induce T-cell metabolic reprogramming (e.g., impaired OXPHOS and reduced mitochondrial quality) and functional exhaustion [87]. This process establishes an “immune escape highway”. Immune evasion mechanisms extend beyond T cells; for example, *galectin-2 (Lgals2)* promotes M2 macrophage polarization via the *CSF1–CSF1R* axis, facilitating mitochondrial transfer and immunosuppression [88].

In summary, TNBC exploits MQC dysregulation through metabolic reprogramming, intercellular mitochondrial trafficking, and immune cell polarization to sustain immune evasion, perpetuating a vicious chemoresistance–immunosuppression cycle. Current research remains fragmented, with unclear clinical translation pathways. Future efforts should integrate multiomics data to map the MQC-immune evasion regulatory network and conduct prospective trials to validate strategies targeting this axis, potentially breaking the resistance-immune escape synergy in TNBC.

#### 4.1.3. Metastasis and Stemness Maintenance

TNBC is more aggressive and has a greater metastatic propensity than other breast cancer subtypes [89]. The survival and motility of metastatic cells depend on mitochondrial respiration and OXPHOS, suggesting that mitochondrial-targeted therapies could exploit this dependency to improve TNBC prognosis [90]. EMT, a critical process conferring metastatic and invasive capacities, involves E-cadherin downregulation and mesenchymal marker upregulation. Aberrant E-cadherin promoter methylation disrupts the regulation of cellular motility in TNBC [91]. Reduced *miR-770-5p* expression is correlated with TNBC proliferation and metastasis. Fei et al. [92] demonstrated that by using the antimicrobial peptide merecidin to act on the cytoplasm of tumor cells in nude mice, increasing the expression of *miR-770-5p* can negatively regulate vimentin, thereby delaying the metastasis and proliferation of breast cancer cells. Mitochondrial dysfunction is linked to EMT progression, with *SIRT1* modulating mitochondrial biogenesis and energy metabolism to regulate EMT outcomes [9]. As a deacetylation substrate of *SIRT1*, *PGC-1α* activates the transcription factors *NRF-1/NRF-2* to control *TFAM* and *TFB1M* expression, driving mitochondrial biogenesis. Studies have shown that *PGC-1α* overexpression promotes TNBC metastasis via the *NRF-1/TFAM* axis [93]. Unlike *ER*+ breast cancers, TNBC displays glycolytic preference and reduced OXPHOS activity owing to oncogenic mutations, mitochondrial defects, transcriptional dysregulation and hypoxic–nutrient-deprived microenvironments [94]. This metabolic heterogeneity, exemplified by the Warburg effect [30], enables rapid tumor growth under nutrient stress. In some TNBC cases, stromal cells exhibit glycolytic activity to supply lactate to tumor cells (reverse Warburg effect), with low *GLUT1/MCT4* in tumor cells, high *MCT4* in the stroma, and elevated *LDHA* driving metastasis [95].

TNBC cells maintain stem-like properties through specific mechanisms, preserving undifferentiated states to form new tumor foci and drive recurrence. Residual CSCs post-treatment contribute to poor prognosis and involve multiple signaling pathways [96,97]. Patients with chemoresistant TNBC exhibit *MYC/MCL1* coamplification. Lee et al. [10] reported that *siRNA*-mediated MYC/MCL1 knockdown reduces *ALDH*+ cell formation, mitochondrial respiration (OCR), ROS levels, and CD44high/CD24low populations in MDA-MB-436 and SUM159PT cells, whereas overexpression enhances stemness. *ALDH*+ cells, potential CSC targets, drive TNBC progression [98]. KK-LC-1 (CT83/Cxorf61), which is overexpressed in TNBC tissues, is correlated with migration, invasion, and wound-healing capacities [99]. Bu et al. [100] revealed that KK-LC-1 maintains stemness via the *FAT1-Hippo-YAP1/SOX*2 axis, promoting FAT1 ubiquitination to activate *SOX2* and *ALDH1A1* transcription. Mitochondrial quality control factors interact with *ALDH*+ expression to influence TNBC progression. Epidermal growth factor (EGF) phosphorylates DRP1 via the ERK/AMPK pathway in TNBC cells. DRP1 upregulation correlates with the ALDH+ cell proportion, OXPHOS, and FAO, fueling CSC energy demands [101]. Activated DRP1 induces mitochondrial fission, driving metabolic reprogramming, ROS modulation, and stemness-mediated metastasis [102]. DRP1 elevation coincides with SOD2 upregulation, which scavenges excess ROS to sustain CSCs and promote EMT [103]. Mitochondrial fission-induced ROS activate the NF-κB and HIF-1α pathways, increasing the expression of stemness genes (e.g., *OCT4 and NANOG*) [104]. Xu et al. [105] reported that hypoxia-induced HIF-2α is critical for TNBC progression, where HIF-2α upregulation promotes miR-141-mediated *MALAT1* suppression, impairing autophagy and metastasis.

**Table 1:** MQCs drive the malignant phenotype of TNBC.

Mechanism	Function	Author	References
Chemotherapy resistance	Drug efflux; relatively quiescent CSCs; hypoxic microenvironment; evasion of apoptosis; unique *miRNA* expression profile; intratumoral heterogeneity in TNBC.	Nedeljković M.	[5]
	Mitochondria-driven metabolic reprogramming promotes tumor survival and disease progression, and cancer stem cells in TNBC tend to favor OXPHOS metabolism.	Missiroli S.	[10,78,79]
	MYC and MCL are enriched in drug-resistant TNBC cells, and they enhance mitochondrial OXPHOS, activate the hypoxia pathway, and mediate drug-resistant characteristics.	Lee K.M.	[10]
	MBD2c activates TFAM through *SIRT3*-mediated deacetylation, thereby upregulating the transcription of *mtDNA* and mitochondrial respiration. Meanwhile, inhibiting MBD2c can significantly enhance the sensitivity to cisplatin.	Hao Y.	[80]
Immune evasion	Cells treated with metformin and 2-deoxy-D-glucose show mitochondria with normal morphology and improved quality under electron microscopy. Glycosylated *PD-L1* in these cells is significantly inhibited, and they exhibit potent anti-tumor efficacy.	Repas J.	[85]
	In paclitaxel-resistant TNBC cells, the expression of *ATAD3A* is upregulated, inhibiting mitophagy and leading to a decrease in mitochondrial *PD-L1*, which enhances immune evasion.	Xie X.Q.	[86]
	Cancer cells in TNBC induce metabolic reprogramming and functional exhaustion of T cells through a bidirectional mitochondrial transfer mechanism.	Ikeda H.	[87]
	Lgals2 can regulate the CSF1/CSF1R axis to induce M2 polarization of macrophages, promoting mitochondrial transfer and immune evasion.	Ji P.	[88]
Metastasis and stemness maintenance	EMT reduces E-cadherin, and the abnormal methylation of the E-cadherin promoter affects the regulation of tumor cell motility and invasiveness.	Noyan S.	[91]
	Using the antimicrobial peptide merecidin to increase the expression of *miR-770-5p* can negatively regulate vimentin, thereby delaying the metastasis and proliferation of breast cancer cells.	Ma F.	[92]
	*SIRT1* controls the outcome of EMT by regulating mitochondrial biogenesis and energy metabolism.	Zhang J.	[9]
	*PGC-1α* is deacetylated and activated by *SIRT1*, and it binds with the factors NRF-1 and NRF-2 to regulate the expression of genes such as *TFAM* and *TFB1M*, affecting the process of mitochondrial biogenesis. When *PGC-1α* is highly expressed, it drives OXPHOS, thereby promoting the metastasis of TNBC.	Fan S.	[93]
	TNBC metabolism is characterized by a preference for glycolysis and low OXPHOS activity, a shift associated with tumor metabolic reprogramming and the tumor microenvironment.	Wang Z.	[94]
	Low *GLUT1/MCT4* in tumor cells, high *MCT4* in stromal cells, and high expression of *LDHA* play a key role in the reverse Warburg effect, which is of great significance for the reversal of TNBC metastasis.	Cheng S.Y.	[95]
	Reducing the expression of MYC and MCL1 via *siRNA* increases the formation of *ALDH*+ cells.	Lee K.M.	[10]
	*ALDH*+ cells, as potential targets for CSC-directed therapy in TNBC, can intervene in the development process of TNBC through various mechanisms.	Liu C.	[98]
	KK-LC-1 is highly expressed in breast tissue and is significantly associated with the migration, invasion, and scratch healing abilities of TNBC cells.	Zhu X.	[99]
	KK-LC-1 promotes the ubiquitination and degradation of FAT1, activates the transcription of *SOX2* and *ALDH1A1*, thereby enhancing the self-renewal and invasive capacity of *ALDH*+ stem cells.	Bu J.	[100]
	EGF phosphorylates DRP1, and high expression of DRP1 is positively correlated with the proportion of *ALDH*+ cells, OXPHOS, and FAO levels, providing energy for CSCs.	Weiner-Gorzel K.	[101]
	Activated DRP1 induces the mitochondrial fission process, promoting the stemness and metastasis of TNBC through metabolic reprogramming and ROS regulation. Meanwhile, the increase in DRP1 is accompanied by upregulation of SOD2, which can clear excessive ROS and maintain the survival of CSCs.	Shome R.	[102,103]
	The mitochondrial fission process leads to increased ROS levels, stabilizes HIF, and simultaneously activates the NF-κB and HIF-1α pathways, upregulating stemness genes such as *OCT4* and *NANOG*, thereby promoting transcription.	Altea-Manzano P.	[104]
	Hypoxia induces the upregulation of HIF-2α expression, thereby promoting miR-141 to target and bind to *MALAT1*, inhibiting its mediation of cell autophagy, degradation, and metastasis.	Xu F.	[105]

### 4.2. Therapeutic Strategies for TNBC Targeting Mitochondrial Quality Control

**Figure 3:**
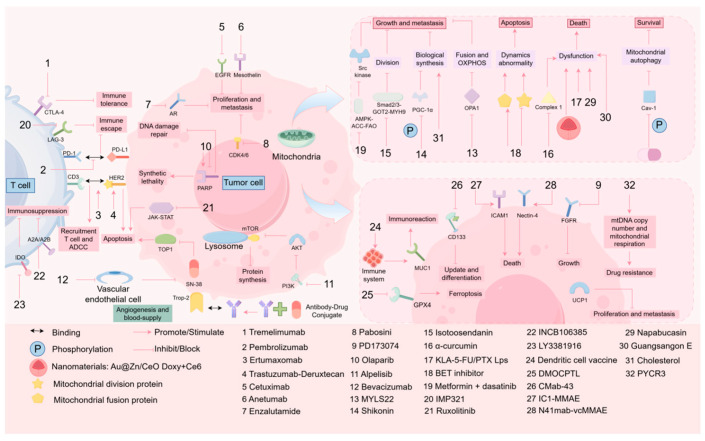
Drug-target mechanism map for the treatment of TNBC. This schematic diagram integrates the multiple types of targets involved in the treatment of TNBC and the corresponding drugs and mechanisms of action. The diagram shows the following core elements: drugs can release the immune escape mechanism of tumor cells and enhance the capture and killing of tumor cells by immune cells by acting on relevant targets on tumor cells or T cells; at the same time, drugs can also inhibit the growth, proliferation and differentiation of tumor cells by altering the metabolic pathway of tumor cells or the process of genetic material expression. The figure was created using the FigDraw (https://www.figdraw.com/static/index.html#/paint_user_home, Export date: 16 June 2025) platform.

The relentless pursuit of effective therapeutic strategies for TNBC continues, with MQC-targeted approaches emerging as a promising frontier in the management of this recalcitrant malignancy. Accumulating evidence highlights the therapeutic potential of several agents and novel targets, offering new possibilities for improving TNBC treatment outcomes and prognosis (Figure 3 and Table 2).

#### 4.2.1. Available Drug Regimens

Multiple clinical or experimental drugs have demonstrated efficacy in MQC-targeted TNBC therapy. Park et al. [106] revealed that FAO activates the *Src* oncogenic pathway in TNBC, whereas low-dose metformin potentiates *AMPK–ACC–FAO* signaling to further stimulate *Src* kinase activity. Their study demonstrated that metformin combined with dasatinib (a Src inhibitor) suppresses TNBC tumor growth and metastasis through mitochondrial–oncogene crosstalk. Rossi et al. [107] reported that BET inhibitors induce an imbalance in mitochondrial dynamics by downregulating fission proteins and upregulating fusion proteins, thereby promoting TNBC cell apoptosis. Chen et al. [108] developed KLA-modified liposomes coloaded with 5-fluorouracil and paclitaxel (KLA-5-FU/PTX Lps), which exhibited robust mitochondrial targeting, induced severe mitochondrial dysfunction, and triggered apoptosis in TNBC-bearing murine models. Zhang et al. [109] reported that isotoosendanin (ITSN) promotes *MYH9* degradation via the *Smad2/3–GOT2–MYH9* axis, inhibiting MYH9-mediated mitochondrial fission and pseudopod formation to suppress TNBC metastasis. Baek et al. [59] demonstrated that *DNA*-damaging chemotherapy elevates OPA1 levels, driving mitochondrial fusion and oxidative phosphorylation. Subsequent OPA1 inhibition using *MYLS22* suppressed residual TNBC cell regrowth, providing insights into overcoming chemoresistance via MQC modulation. The above research summaries all focus on the midstream and upstream links of the MQC mechanism. However, as downstream responses secondary to mitochondrial dysfunction, such as the clearance of accumulated ROS and damaged mitochondria, what mechanisms could serve as new targets? In the research of related drugs, some scholars have found that Napabucasin, as a *STAT3* inhibitor, can significantly enhance the sensitivity to paclitaxel by inhibiting the *STAT3* signaling pathway and impairing mitochondrial function (such as reducing the activity of complex I and ATP levels), which leads to ROS accumulation. This study also provides a feasible solution for addressing the problem of chemotherapy resistance in TNBC [81]. In addition, the natural compound GSE isolated from mulberry has been proven to promote ROS accumulation and inhibit TNBC cell growth by inducing mitochondrial dysfunction and mitophagy [110].

#### 4.2.2. Traditional Chinese Medicine and Novel Therapies

Natural product-derived compounds exhibit remarkable potential in MQC-targeted TNBC therapy. Cruz et al. [111] reported that α-mangostin reduces mitochondrial complex I activity and mitochondrial mass, inducing lethal mitochondrial dysfunction in TNBC cells. Wang et al. [112] demonstrated that astragaloside IV (As) enhances mitochondrial biogenesis and activity in tumor-infiltrating lymphocytes (TILs), whereas oxymatrine promotes TIL infiltration into tumors, synergistically ameliorating the immunosuppressive tumor microenvironment. Falcone et al. [113] proposed that high-dose resveratrol disrupts mitochondrial function via long-chain acylcarnitine accumulation, suggesting its utility in targeting aggressive breast cancers. Hong et al. [114] revealed that the ginsenoside Rk1 induces ROS generation, inhibits the PI3K/Akt pathway, and reduces the mitochondrial membrane potential, exerting potent anti-TNBC activity with minimal organ toxicity. Chen et al. [115] engineered a pH-sensitive AS1411-triptolide conjugate (AS-TP) that selectively activates mitochondrial apoptosis pathways in TNBC cells while mitigating systemic toxicity, highlighting its dual advantages in efficacy and safety.

Nanotechnology-based drug delivery systems have revolutionized MQC-targeted therapeutic approaches. Xiao et al. [116] developed doxycycline (Doxy)- and chlorin e6 (Ce6)-coloaded nanoparticles, where Doxy-induced mitochondrial dysfunction enhances Ce6-mediated photodynamic therapy, suppresses autophagic flux, and upregulates MHC-I expression to promote antigen presentation and CTL activation. Zhang et al. [117] constructed Au@Zn/CeO (AZC)-engineered *Escherichia coli* for targeted delivery to CSC-enriched TNBC regions. The AZC system induces mitochondrial dysfunction, ROS overproduction, and photothermal ablation, effectively eliminating CSCs. These findings validate the feasibility of MQC-targeted nanotherapeutics in TNBC treatment, with CSC-specific mitochondrial targeting emerging as a promising direction [118].

#### 4.2.3. Therapeutic Targets of TNBC by Targeting MQC

Novel MQC-related therapeutic targets have been identified. In the preceding text, we mentioned that overexpression of *PGC-1α* can promote the metastasis of TNBC tumor cells. Regarding this, Fan et al. [93] demonstrated that cancer-associated fibroblasts (CAFs) activate the *PGC-1α/ERRα* axis to increase mitochondrial biogenesis in TNBC cells. Shikonin (an extract from the root of the lithospermum plant) inhibits this process via *PGC-1α* phosphorylation, reducing mitochondrial quantity and ATP production to suppress metastasis. In addition, Faeghh et al. [119] discovered through experiments that cholesterol can induce the high expression of ERRα and enhance its targeting function, as well as strengthen its interaction with *PGC-1α*. This process has a stimulating effect on the transcription, proliferation, and metastasis of TNBC cells. Therefore, reducing the cholesterol levels in TNBC patients may have a positive impact on their prognosis. Jiang et al. [120] revealed that phosphorylated caveolin-1 (Cav-1) suppresses mitophagy initiation in a phosphorylation-dependent manner, leading to damaged mitochondrial accumulation and mtROS overproduction in MDA-MB-231 cells, suggesting that Cav-1 phosphorylation is therapeutically vulnerable. Ding et al. [121] established a TNBC prognostic model based on nine *mitochondrial autophagy-related genes (MRGs)*, including *MRPS5 and PYCR1*, where elevated MRG expression was correlated with reduced patient survival. Regarding prognosis, as mentioned earlier, stem cells, as one of the key factors of chemoresistance, the further revelation of the close association between mitochondrial metabolic remodeling and stem cell enrichment in the aforementioned studies is also a key mechanism of TNBC chemoresistance. Ioannis S Pateras et al. [122] found that short-term starvation can make TNBC cells more sensitive to chemotherapy. Mechanistically, in the short-term starvation and chemotherapy group, the expression of *mtDNA* in tumor cells was downregulated, which in turn inhibited mitochondrial biogenesis, damaged mitochondrial respiration, and altered their metabolic profiles, causing ROS accumulation and upregulating the sensitivity of tumor cells to chemotherapeutic drugs [122]. Although this study is currently limited to in vitro and in vivo experiments, it is expected to bring new treatment options for clinical TNBC in the future and improve the long-term prognosis of TNBC. Feifei Zhuang et al. [123] found that pyrroline-5-carboxylate reductase 3 (PYCR3) is significantly upregulated in TNBC tumor tissues, and it promotes drug resistance by increasing *mtDNA* copy number and mitochondrial respiration. Knockdown of PYCR3 has been proven to reverse the acquired resistance to the chemotherapeutic drug doxorubicin. This study provides a new therapeutic target for solving the problem of chemoresistance in TNBC. Uncoupling protein 1 (UCP1), a member of the mitochondrial anion carrier protein (MACP) family, plays an important role in the regulation of membrane potential and lipid metabolism balance in the occurrence and development of various cancers [124,125]. Studies have found that [126] UCP1 can increase the expression of PINK1 and Parkin proteins, regulate the autophagy and apoptosis of mitochondria, and at the same time impair the pro-invasive effect of the pyroptosis protein GSDMB on TNBC, implying its positive role in inhibiting the proliferation and metastasis of TNBC.

In conclusion, MQC-targeted strategies, spanning mitochondrial biogenesis regulation, dynamic modulation, and mitophagy manipulation, provide innovative avenues for TNBC therapy. The optimization of validated agents through improved formulations, combination regimens, and nanotechnology-enhanced delivery systems holds promise for enhancing efficacy while minimizing toxicity. Future research should prioritize elucidating the mechanistic roles of MQC regulators in TNBC pathogenesis and translating these discoveries into clinical applications. The integration of advanced drug delivery platforms with mitochondrial-targeted therapies may overcome treatment resistance and improve patient outcomes.

**Table 2:** Mitochondria-related therapeutic targets.

Research Strategy	Mechanism of Action	Drug/Target	Research Model	Key Findings
Regulation of mitochondrial metabolism	Activates the *AMPK-ACC-FAO* signaling pathway and inhibits the Src kinase pathway [106]	Metformin + Dasatinib	TNBC mouse model	Suppresses tumor growth and metastasis
	Reduces mitochondrial complex I activity, inducing mitochondrial dysfunction [111]	α-Curcumin	TNBC cell lines	Induces cancer cell death
	Inhibition of the *STAT3* signaling pathway and impairment of mitochondrial function (such as reducing the activity of complex I and ATP levels) [81]	Napabucasin	TNBC cell model	Significantly enhance the sensitivity to paclitaxel
Mitochondrial dynamics regulation	Inhibits MYH9-regulated mitochondrial fission [109]	Isotoosendanin (ITSN)	TNBC metastasis model	Inhibits tumor cell metastasis
	Inhibits mitochondrial fission, promotes fusion, causing dynamic abnormalities [107]	BET inhibitors	TNBC cellular models	Promotes tumor cell apoptosis
	Inhibit OPA1-mediated mitochondrial fusion [59]	OPA1 inhibitor (MYLS22)	Chemotherapy-resistant TNBC model	Inhibit the regrowth of residual tumors
Nanodrug delivery system	Disrupts mitochondrial function, inhibits autophagy, enhances antigen presentation [108]	KLA-5-FU/PTX liposomes	Human breast cancer mouse model	Exhibits significant antitumor activity
	Photodynamic therapy combined with mitochondrial dysfunction [116]	Doxy/Ce6 nanodrugs	Tumor models	Enhances photodynamic efficacy and promotes immune recognition
	Induces mitochondrial dysfunction, ROS accumulation, and photothermal therapy [117]	Au@Zn/CeO engineered bacteria	TNBC stem cell-enriched models	Eliminates CSCs
Mitochondrial biogenesis targeting	Inhibits the *PGC-1α/ERRα* axis, reducing mitochondrial biogenesis [93]	Shikonin (Lithospermum extract)	TNBC cell metastasis model	Suppresses ATP production and tumor metastasis
Mitophagic regulation	Increase the expression of PINK1 and Parkin proteins, while impairing the pro-invasive effect of the pyroptosis protein GSDMB on TNBC [126]	UCP1	TNBC mouse model	Has a positive effect on inhibiting the proliferation and metastasis of TNBC
	Phosphorylates Cav-1 to restrict mitophagy, leading to damaged mitochondrial accumulation [120]	Cavity protein-1 (Cav-1) phosphorylation	TNBC cell model	Increases mtROS and suppresses cancer cell survival
	Induce mitochondrial dysfunction and mitophagy, promoting ROS accumulation	GSE	Human breast cancer mouse model	Inhibit TNBC cell growth
Prognostic models and gene targets	Mitochondrial autophagy-related genes (MRGs) expression correlates with prognosis [121]	*MRPS5, PYCR1, C20orf27 (9 MRGs)*	TNBC patient cohort	High MRG expression significantly reduces patient survival rates
	Increase *mtDNA* copy number and mitochondrial respiration to promote drug resistance [124]	PYCR3	TNBC cell metastasis model	Knockdown of PYCR3 can reverse the acquired resistance of TNBC to the chemotherapeutic drug doxorubicin.

## 5. Summary and Outlook

TNBC represents the most aggressive breast cancer subtype with a dismal prognosis, posing significant therapeutic challenges because of the absence of effective molecular targets, high heterogeneity, robust invasiveness, and low survival rates. Recent advances highlight the pivotal role of MQC in TNBC pathogenesis. Elucidating the interplay between MQC dysregulation and TNBC malignancy is critical for unraveling molecular mechanisms and developing novel therapeutic strategies. Mitochondria, as central hubs of cellular energy metabolism and signaling platforms, directly influence cell survival and proliferation. In TNBC, MQC dysfunction drives malignant progression through multiple pathways: aberrant mitochondrial fission activates the AMPK–EMT pathway to increase metastatic potential; defective mitophagy contributes to chemoresistance; dysregulated biogenesis reprograms metabolic networks for energy acquisition; and imbalanced dynamics disrupt the immune microenvironment, fostering immune tolerance. These observations underscore the central role of MQC in TNBC biology.

Current therapeutic strategies targeting MQC have gained momentum. Agents such as metformin combined with Src inhibitors, α-curcumin, and BET inhibitors suppress TNBC growth via distinct mechanisms. Concurrently, advancements in nanodrug delivery systems, including drug-loaded nanoparticles and engineered bacteria-based carriers, have demonstrated promising antitumor efficacy in preclinical models. However, clinical translation faces challenges: the complexity and heterogeneity of MQC regulatory networks necessitate deeper mechanistic exploration; the efficacy of MQC-targeted drugs requires validation in large-scale clinical trials; and the molecular diversity of TNBC demands biomarker-guided precision therapies.

Future research should prioritize (1) identifying novel MQC-related therapeutic targets, (2) developing mitochondrial-targeted molecules and nanocarriers, (3) optimizing combination therapies to overcome monotherapy limitations, and (4) accelerating translational studies to bridge preclinical and clinical gaps.

In conclusion, MQC plays a central role in TNBC progression. Targeting MQC pathways offers transformative potential for TNBC treatment. Continued research and innovation in this field may substantially improve patient outcomes and survival.

## Data Availability

Not applicable.
